# Tunable invisibility cloaking by using isolated graphene-coated nanowires and dimers

**DOI:** 10.1038/s41598-017-12413-4

**Published:** 2017-09-22

**Authors:** Mahin Naserpour, Carlos J. Zapata-Rodríguez, Slobodan M. Vuković, Hamid Pashaeiadl, Milivoj R. Belić

**Affiliations:** 1 Department of Optics and Optometry and Vision Science, University of Valencia, Dr. Moliner 50, 46100 Burjassot, Spain; 2 Department of Physics, College of Science, Shiraz University, Shiraz, 71454 Iran; 3 Center of Microelectronic Technologies, Institute of Chemistry, Technology and Metallurgy (IHTM), University of Belgrade, Njegoševa 12, 11000 Belgrade, Serbia; 4 Texas A&M University at Qatar, P.O. Box 23874 Doha, Qatar

## Abstract

We investigate, both theoretically and numerically, a graphene-coated nano-cylinder illuminated by a plane electromagnetic wave in the far-infrared range of frequencies. We have derived an analytical formula that enables fast evaluation of the spectral window with a substantial reduction in scattering efficiency for a sufficiently thin cylinder. This polarization-dependent effect leads to tunable resonant invisibility that can be achieved via modification of graphene chemical potential monitored by the gate voltage. A multi-frequency cloaking mechanism based on dimer coated nanowires is also discussed in detail.

## Introduction

Recent progress in design and manufacturing of new photonic materials has enabled novel models and multifunctional devices to achieve unprecedented control of light. In particular, by using various techniques based on transformation optics, the possibilities have appeared to electromagnetically isolate a space region for certain frequencies or range of frequencies. It means that an object located inside such a space region will practically stop interacting with the illuminating light^1–3^. In fact, a remote observer will not be able to detect the presence of the objects that are shielded and protected by optical cloaking. The materials needed for practical applications of these techniques, either artificial or natural, require extremal values of their permittivity and/or permeability. However, coatings can be designed such as metal-dielectric multilayer or metasurfaces to drastically reduce the scattered signal from subwavelength particles and make the assembly nearly undetectable^4–7^. Of course, light interacts with the nanoparticles, but the destructive interference at the different elements that constitute the complex nanostructure practically cancels out the scattered radiation.

Graphene represents a groundbreaking and exciting material that combines suitable characteristics for the use in optoelectronic devices in far-infrared and THz region of frequencies due to the intrinsic ability to mold the surface current with low-loss rates^8,9^. High conductivity within atomically thin layer of graphene, and significant tuning ability via the applied bias voltage, enables the cancellation of scattering effects. Drastically reduced overall visibility of the scattering object that was conducted via graphene monolayer wrapping the cylinder, for the first time, was extended to the far-infrared range of frequencies by Chen and Alu^10^. In that case, the local polarizability of a nanowire with moderately wide diameter and the graphene coating sheet with the opposite signs can be mutually cancelled under suitable layout. Different schemes have been proposed with plasmonic compounds, using a dipole moment of opposite phase to attain a scattering cancellation^11–13^. If the incident radiation is *p*-polarized, however, the spectral response of the scattering cross section seems to be totally dissimilar^14^. Then, a set formed by a peak, associated with the scattering via a narrow Mie mode, and a valley offers the prospect to obtain invisibility with unprecedented control over the wire resonance spectral location by changing the chemical potential.

In the present paper we extend the previous concept by employing a *p*-polarized incident plane wave that illuminates a graphene coated dielectric nanowire in order to get near the fundamental localized surface plasmon polariton. Subsequently we optimize the scattering cancellation of the nanocavity. The proposed theoretical model is critically discussed with a conclusion that a characteristic lineshape appears comparable to the shape of Fano resonance^15^. The check of validity and estimation of our results is performed by using the full-wave Lorenz-Mie method^16^.

## Reducing the scattering efficiency

**Figure 1:**
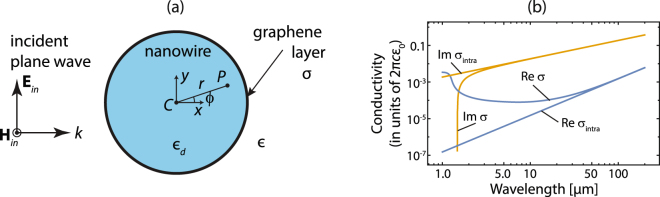
(**a**) Illustration of the atomically thin graphene layer of surface conductivity *σ* coating a nano-sized scattering cylinder of relative permittivity *ε*_*d*_, where the environment medium has a permittivity *ε*. Here we consider an incident TE^*z*^-polarized plane wave. (**b**) Real and imaginary part of the surface conductivity for graphene at *T* = 300 K with *μ* = 0.5 eV and Γ = 0.1 meV. The surface conductivity is given in units of 2*πcε*_0_ = 1.67 × 10^−2^ S. We also include *σ*_intra_ representing the Drude model applied to graphene, which is valid in the far infrared and beyond.

Let us consider a cylindrical dielectric nanowire of radius *R* and relative permittivity *ε*_*d*_ over-coated by an atomically thin graphene shell, and oriented along the *z* axis, as illustrated in Fig. 1(a). The nanowire is assumed to be placed in a lossless environment medium of the relative permittivity *ε* and illuminated by a plane electromagnetic wave that propagates along the *x* axis with the electric field vector **E**_*in*_ in the *xy* plane. The graphene conductivity *σ* within the coating shell is described in the local random phase approximation by using Kubo formula^17^ as a sum of intraband *σ*_intra_ and interband *σ*_inter_ contribution, where

1 $${\sigma }_{{\rm{intra}}}=\frac{2i{e}^{2}{k}_{B}T}{\pi {\hslash }^{2}(\omega +i{\rm{\Gamma }})}\,\mathrm{ln}[2\,\cosh (\frac{\mu }{2{k}_{B}T})],$$

and

2 $${\sigma }_{{\rm{inter}}}=\frac{{e}^{2}}{4\hslash }[\frac{1}{2}+\frac{1}{\pi }\arctan (\frac{\hslash \omega -2\mu }{2{k}_{B}T})-\frac{i}{2\pi }\,\mathrm{ln}\,\frac{{(\hslash \omega +2\mu )}^{2}}{{(\hslash \omega -2\mu )}^{2}+{(2{k}_{B}T)}^{2}}]\mathrm{.}$$

Here, −*e* is the charge of an electron, $\hslash$ is the reduced Planck’s constant, *k*_*B*_ is Boltzmann’s constant, *T* is the temperature, Γ is the charge carriers scattering rate, *μ* is the chemical potential and $\hslash$ *ω* is the photon energy. In Fig. 1(b) we show the graphene conductivity at *T* = 300 K with *μ* = 0.5 eV and Γ = 0.1 meV. Also, the time harmonic dependence of the field is adopted to be of the form *exp*(−*iωt*).

To estimate analytically the scattering efficiency of the coated nanowire, we followed the Lorenz-Mie scattering method given in detail for instance in refs^10,14,18–20^. Details on this well-established technique can be found in our Methods section. The scattering coefficients *a*_*n*_, given by Eq. (22), enable the fast estimation of the scattering efficiency^16^:

3 $${Q}_{sca}=\frac{2}{kR}\sum _{n=-\infty }^{+\infty }|{a}_{n}{|}^{2}\mathrm{.}$$

**Figure 2:**
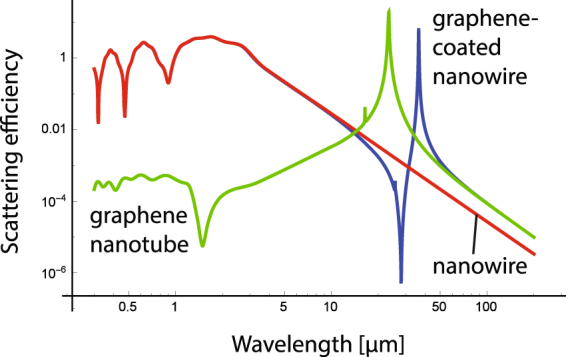
Scattering efficiency, *Q*_*sca*_, of a bare cylinder of radius *R* = 0.5 *μ*m and permittivity *ε*_*d*_ = 3.9 immersed in air (*ε* = 1), a graphene nanotube of chemical potential *μ* = 0.5 eV surrounded by air, and the graphene-coated nanocylinder. In all calculations we set *T* = 300 K and Γ = 0.1 meV.

Typically, the terms with low order *n* effectively contribute to the summation when the size of the scattering object is small enough. The invisibility condition is achieved when the scattering coefficients *a*_*n*_ simultaneously tend to zero. In contrast, resonances can be attributed to the pole(s) of scattering coefficient(s). Now, we would like to compare the scattering efficiency of: (i) a bare dielectric cylinder, (ii) a graphene nanotube, and (iii) a uniform-graphene coated dielectric nanocylinder. We assume the dielectric core made of SiO_2_ with the relative permittivity *ε*_*d*_ = 3.9 and radius *R* = 0.5 *μ*m; the graphene monolayer with the chemical potential *μ* = 0.5 eV; ambient temperature *T* = 300 K and carrier scattering rate Γ = 0.1 meV. The last two parameters are kept fixed in all numerical calculations that follow. The scattering efficiency spectrum *Q*_*sca*_(*λ*), where *λ* is the wavelength of the TE^*z*^-polarized illuminating radiation, for each of the three objects is presented in Fig. 2. In the range of long wavelengths, the curve pattern is formed by a peak resonance at *λ* = 36.4 *μ*m and a minimum of scattering efficiency at a wavelength of 28.1 *μ*m. Note that the ratio *R*/*λ* = 1.78 × 10^−2^ when scattering is effectively canceled, confirming that the size of the object to become electromagnetically undetectable is small compared to the operative wavelength. The resonance wavelength and the invisibility wavelength can be found in the vicinities of the resonance peak for the free-standing graphene nanotube, found at *λ* = 23.4 *μ*m. Without the presence of the graphene coating, the dielectric cylinder cannot exhibit any significant feature of the scattering efficiency in such spectral range. One might presume that the set of peak and valley patterned in the scattering efficiency follows the characteristic lineshape of the Fano resonance, where the emission of electromagnetic waves by the coated nanowire creates the interference between the nonresonant scattering from the dielectric core and scattering by narrow Mie modes in the graphene nanotube^21^. The graphene-coated nanowire exhibits a scattering efficiency dominated by the graphene nanotube (surrounded by air) at long wavelengths, where the graphene coating isolates the central dielectric cylinder. On the other hand, the bare (uncoated) nanowire dominates the scattering properties for shorter wavelengths, due to its stronger response far from the Rayleigh scattering regime. Note that in Fig. 2, additional minima of the scattering efficiency can be found in the visible and near-IR due to the contribution of high-order Fano resonances, as described in refs^22,23^.

It is necessary to point out that evaluation of the electromagnetic field components, given by (14–17), as well as the scattering efficiency (3), can be alternatively achieved by using a matrix method to derive the scattering coefficients *a*_*n*_ and *b*_*n*_^24–26^. In this respect, the finite width of the graphene layer (*t*_*g*_) has to be introduced, that leads to the corresponding graphene permittivity *ε*_*g*_ = *iσ*/*ε*_0_ *ωt*_*g*_. In particular, *t*_*g*_ = 0.5 nm has been employed, where the calculated scattering efficiency for multilayered nanotubes (not shown here) is in excellent agreement with the results depicted in Fig. 2.

**Figure 3:**
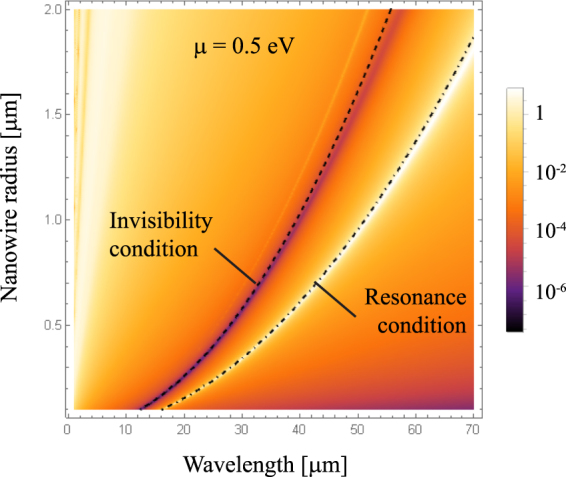
Scattering efficiency spectra, *Q*_*sca*_, for a uniform-graphene coated cylinder illuminated by a TE^*z*^-polarized plane wave, when the radius *R* varies, maintaining fixed *ε*_*d*_ = 3.9 and *μ* = 0.5 eV. We included analytical equations (7) and (8) indicating the resonance condition and the invisibility condition, respectively.

The presence of a peak resonance for a graphene nanotube might be behind the Fano-like resonance in the complex graphene-dielectric nanowire. Under such perspective, tuning the resonance peak of the graphene nanotube will enable to shift the invisibility wavelength on demand. This procedure can be carried out by simply modifying the radius of the graphene nanotube^27^. Scattering efficiency for a graphene-coated dielectric nanowire as a function of radius *R* is presented in Fig. 3. It is evident that the peak-valley lineshape is preserved, but shifted towards the longer wavelengths when the radius of the cylinder *R* increases.

On the other side, in order to obtain an analytical expression for the fast evaluation of the invisibility window we use the following approximate expression for *σ*_intra_ that dominates the contribution to graphene conductivity for moderate and low frequencies ($\hslash \omega  < \mu$), and large doping ($\mu \gg {k}_{B}T$):

4 $${\sigma }_{{\rm{intra}}}=\frac{i{e}^{2}\mu }{\pi {\hslash }^{2}(\omega +i{\rm{\Gamma }})}\,(\approx \sigma ),$$

as illustrated in Fig. 1(b). In fact, such expression represents the generalization of the well known Drude model for application to graphene. For example, that model is particularly accurate for the imaginary part of graphene conductivity for *λ* > 10 *μ*m, if all the other relevant parameters stay the same as in Figs 2 and 3. In addition, the requirement $\omega \gg {\rm{\Gamma }}$ (or $\lambda \ll 12.4\,{\rm{mm}}$) gives the upper limit on the wavelength. Therefore, for the parameters used in the present paper, we may conclude that the expression:

5 $${\rm{Im}}(\sigma )\approx \frac{{e}^{2}\mu }{\pi {\hslash }^{2}\omega },$$

can be used accurately. On the other hand, analytical approximations for the resonance wavelength and the invisibility wavelength can be deduced by means of the electrostatics approximation^16,27^. By comparing the squared modulus of the scattering coefficients *a*_*n*_ for nanosized graphene-coated cylinders, one can observe that the first order dominates over the rest of the orders in the spectral range of interest. For $|{a}_{1}{|}^{2}$ and *R* = 0.5 *μ*m, we observe a maximum at 36.4 *μ*m and a minimum at 28.1 *μ*m, two features which are replicated in the scattering efficiency. In the latter, in fact an additional peak is also observed at 25.7 *μ*m corresponding to a resonance peak of $|{a}_{2}{|}^{2}$. Further peaks and minima are difficult to be registered. For that reason, the scattering orders *n* = ±1 can be taken into account, only. Within the electrostatics approximation, we further use a Taylor expansion in the limit *R* → 0, retaining only the term of the lowest order, such as ${J}_{1}(x)\approx x\mathrm{/2}$ and ${H}_{1}^{\mathrm{(1)}}(x)\approx -2i/\pi x$. Then,

6 $${a}_{1}=\frac{i\pi }{4}{(kR)}^{2}\frac{{\varepsilon }_{d}-\varepsilon +i\sigma /{\varepsilon }_{0}\omega R}{{\varepsilon }_{d}+\varepsilon +i\sigma /{\varepsilon }_{0}\omega R}\mathrm{.}$$

In other words, the complex scattering object under consideration behaves like a solid cylinder of dielectric constant ${\varepsilon }_{d}+i\sigma /{\varepsilon }_{0}\omega R$, that assumes a bulk conductivity *σ*/*R*.

When $\omega \gg {\rm{\Gamma }}$, it follows from Eq. (6) that the resonance appears at the frequency:

7 $${\omega }_{{\rm{res}}}=\sqrt{\frac{{e}^{2}\mu }{\pi {\hslash }^{2}{\varepsilon }_{0}R({\varepsilon }_{d}+\varepsilon )}}\mathrm{.}$$

Assuming that *ε*_*d*_ and *ε* are positive, a resonance peak will occur since Im(*σ*) > 0 in the spectral range of interest. Note that the peak resonance is associated to a localized surface plasmon in the graphene-coated cylinder, and its dispersion relation can be interpreted such that this bound mode cyclically propagates along the cylinder perimeter exactly one effective wavelength corresponding to the flat graphene sheet^27^. More interesting in our study, a drastic reduction of the scattering efficiency will be experienced under the constrain ${\varepsilon }_{d}-\varepsilon -{\rm{Im}}(\sigma )/{\varepsilon }_{0}\omega R\,=\,0$. A suitable use of this equation is restricted to frequencies of low losses in graphene (Re(*σ*) < Im(*σ*) occurs at *λ* > 1.53 *μ*m in Fig. 1(b)) and subwavelength particles, $R/\lambda \ll 1$, leading to the condition ${\rm{Im}}(\sigma \mathrm{)/2}\pi c{\varepsilon }_{0}\ll 1$ (in Fig. 1(b) this happens if $\lambda \ll 0.5$ mm) for dielectric cylinders of *ε*_*d*_ > *ε*, evidencing that our invisibility procedure is only valid in the far infrared for practical uses. The frequency for invisibility will appear at

8 $${\omega }_{{\rm{inv}}}=\sqrt{\frac{{e}^{2}\mu }{\pi {\hslash }^{2}{\varepsilon }_{0}R({\varepsilon }_{d}-\varepsilon )}}\mathrm{.}$$

As a consequence, the invisibility window depends on the parameter *μ*/*R*Δ*ε*, where Δ*ε* stands for the difference of dielectric constants between the core and the environment media. In order to meet either the invisibility or the scattering resonance conditions, for given monochromatic field, the nanowire radius *R* has to be adjusted. This is illustrated in Fig. 3, where the analytical expressions (7) and (8) have been included for resonance and invisibility, respectively. Excellent agreement is evident, especially for short radius nanowires. Finally, we would like to emphasise that the scattering efficiency can be accurately calculated as ${Q}_{sca}\,=\,\mathrm{4|}{a}_{1}{|}^{2}/kR$ for sufficiently thin graphene-coated nanowires, that is when the dipolar term given within the electrostatics approximation in Eq. (6) dominates. Therefore, the spectral dependence of the scattering efficiency can be written as

9 $${Q}_{{\rm{sca}}}\propto {\omega }^{3}\frac{{({\omega }^{2}-{\omega }_{{\rm{inv}}}^{2})}^{2}+{(\omega {\rm{\Gamma }})}^{2}}{{({\omega }^{2}-{\omega }_{{\rm{res}}}^{2})}^{2}+{(\omega {\rm{\Gamma }})}^{2}}\mathrm{.}$$

Interestingly, when the regions of invisibility and resonance start to overlap, i.e. when ${\omega }_{{\rm{inv}}}-{\omega }_{{\rm{res}}}\ll \omega$ in the spectral window of interest, *Q*_sca_ approaches the well known Fano resonance lineshape^15^:

10 $${Q}_{{\rm{sca}}}\propto \frac{{(F{\rm{\Gamma }}\mathrm{/2}+\omega -{\omega }_{{\rm{res}}})}^{2}}{{(\omega -{\omega }_{{\rm{res}}})}^{2}+{({\rm{\Gamma }}\mathrm{/2})}^{2}}\mathrm{.}$$

Here, the so called Fano parameter $F=({\omega }_{{\rm{res}}}-{\omega }_{{\rm{inv}}})/({\rm{\Gamma }}\mathrm{/2})$. The validity of such an approximation is limited to the case *ε*_*d*_ ≫* ε*, as discussed below. In the case of anisotropic plasmonic nanotubes such behaviour has been highlighted in ref.^28^.

**Figure 4:**
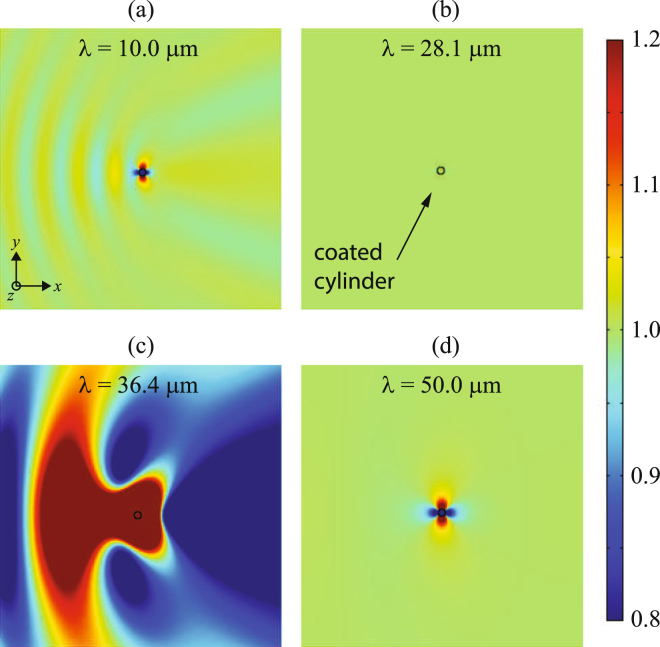
Modulus of the normalized total electric field, |**E**/E_0_|, for a graphene-coated cylinder of radius *R* = 0.5 *μ*m, keeping *ε*_*d*_ = 3.9 and *μ* = 0.5 eV, illuminated by a TE^*z*^-polarized plane wave of wavelength (**a**) *λ* = 10.0 *μ*m, (**b**) *λ* = 28.1 *μ*m coinciding with the invisibility window, (**c**) *λ* = 36.4 *μ*m satisfying the resonance condition, and (**d**) *λ* = 50.0 *μ*m.

In Fig. 4 we show the total electric field inside a coated cylinder, **E**_*d*_, and the field in the enviroment medium, **E**_*in*_ + **E**_*sca*_, normalized to the electric field of the incident plane wave, *E*_0_, at different wavelengths. Out of the spectral band where the invisibility and resonance exist, as illustrated for (a) *λ* = 10 *μ*m, and (d) *λ* = 50 *μ*m, a moderately intense signal that corresponds to a dipolar scattered field is evident. In this case, Rayleigh scattering is governing, which is characterized by a dominant coefficient ${a}_{1}\propto {\lambda }^{-2}$. For $\omega \ll {\omega }_{{\rm{res}}}$ the graphene layer acts as a perfect electric conductor (PEC), and the graphene-coated dielectric cylinder behaves exactly like a bare PEC nanowire. In that case, ${a}_{1}\approx i\pi {(kR)}^{2}\mathrm{/4}$. In contrast, for longer wavelengths graphene conductivity can be neglected and the complex nanowire behaves like an uncoated cylinder. In that case, ${a}_{1}\approx i\pi {(kR)}^{2}({\varepsilon }_{d}-\varepsilon \mathrm{)/4(}{\varepsilon }_{d}+\varepsilon )$. The invisibility window is manifested at the wavelength *λ* = 28.1 *μ*m, where a drastic reduction of the scattering wave field is apparent. Here the first-order scattering coefficient can be straightforwardly approximated by ${a}_{1}\approx -\pi {\omega }_{{\rm{inv}}}{\rm{\Gamma }}{\rm{\Delta }}\varepsilon {R}^{2}\mathrm{/8}{c}^{2}$, and therefore the scattering efficiency increases with the cylinder radius as ${Q}_{{\rm{sca}}}\propto {R}^{3}$ at ${\omega }_{{\rm{inv}}}$. One should note that the electric field is conserved inside and outside the coated nanowire. On the contrary, the resonant condition will be satisfied nearby at *λ* = 36.4 *μ*m, where an enhanced scattered field is emitted by the coated cylinder.

We point out that the invisibility frequency is highly dependent upon the state of polarization of the incident wave field. Provided that the impinging plane wave is TM^*z*^ polarized, that is the magnetic field lies on the *xy* plane, thin coated nanowires will scatter upon a dominant monopolar emission (see Appendix); this happens since the electric field is oriented along the axis of the conducting cylinder. For convenience here we appoint the continuity of the electric field on the boundaries of the particle, which will undergo no variation inside under the electrostatic approximation. This heuristic approach enables us to estimate an effective permittivity of the scatterer including the core and its graphene coating as ${\varepsilon }_{{\rm{eff}}}={f}_{d}{\varepsilon }_{d}+{f}_{g}{\varepsilon }_{g}$, where *f*_*d*_ and *f*_*g*_ are the volume filling factor of the dielectric core and the graphene coating, respectively. Taking into account the lower dimensionality of the graphene layer, we might approach *f*_*d*_ = 1. On the other hand the volume of the graphene layer can be set as

11 $${V}_{g}=[\pi {(R+{t}_{g})}^{2}-\pi {R}^{2}]L,$$

where *L* represents the length of the cylinder. Since ${t}_{g}\ll R$, we may accurately set ${V}_{g}\,=\,2\pi R{t}_{g}L$ leading to the filling factor ${f}_{g}\,=\,2{t}_{g}/R$. As a consequence, we estimate the effective permittivity of the cylinder as ${\varepsilon }_{{\rm{eff}}}={\varepsilon }_{d}+(2{t}_{g}/R)\times (i\sigma /{\varepsilon }_{0}\omega {t}_{g})$. Now it is clear the the invisibility condition will be satisfied when the effective permittivity of the particle coincides with that of the environment medium, that is ${\varepsilon }_{{\rm{eff}}}=\varepsilon$, occurring if ${\varepsilon }_{d}-2{\rm{Im}}(\sigma )/{\varepsilon }_{0}\omega R=\varepsilon$. Such condition formally coincides with that deduced for TE^*z*^ polarized wave fields, except for the fact that the graphene conductivity is doubled. The latter is verified by using the Lorenz-Mie method and subsequently considering the limit ${k}_{0}R\ll 1$ corresponding to ultra-thin wires, as described in detail in the Appendix. To conclude we expect to find a cloaking effect at a frequency ${\omega }_{{\rm{inv}}}^{TM}=\sqrt{2}{\omega }_{{\rm{inv}}}$, that is the invisibility frequency is blue-shifted by a factor $\sqrt{2}$ with respect to the frequency estimated by Eq. (8).

In our study we have considered cylindrical scatterers with an infinite length. In practical cases, the finite length *L* of the graphene-coated cylinder might give rise to unsuitable effects that disturb the demanded reduction of the scattering efficiency. This case has been previously considered in the literature for both metallic and dielectric particles, even including invisible cloaks^29–33^. It has been observed that the drastic minimization of the scattered electromagnetic signal persists for structures where *L* > *λ*. In addition, one expects that the incident wave interacts with the longitudinal boundaries to create guided modes within the structure (graphene plasmons in the coating monolayer and photonic modes in the dielectric core) that propagate along the cylinder axis. Furthermore, Fabry-Perot resonance conditions can be established in case that the length *L* of the cylinder matches a multiple half-wave of the characteristic modal wavelength, causing numerous peaks in the lineshape of the scattering efficiency spectrum by means of wave leakage at the ends. Such Fabry-Perot resonances could be more pronounced using plane wave illumination at oblique incidence, which may even lead to Fano resonances caused by the interplay of these longitudinal guided modes with broadband Mie resonances^34^. Finally, this rich phenomenology might also be further exploited for invisibility applications.

## Tuning invisibility

**Figure 5:**
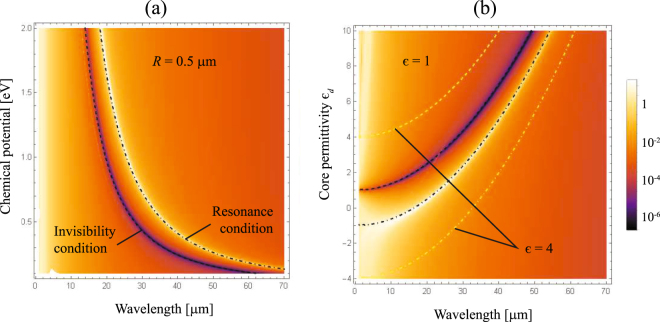
Contour plot of *Q*_sca_ for graphene coated cylinders illuminated by a TE^*z*^-polarized plane wave, when (**a**) The chemical potential *μ* is varied, keeping *ε*_*d*_ = 3.9 and *R* = 0.5 *μ*m, and (**b**) the permittivity *ε*_*d*_ of the dielectric core varies and the environment medium has a dielectric constant ε = 1, maintaining fixed *R* = 0.5 *μ*m and *μ* = 0.5 eV. We also represent the curve of resonance given by Eq. (7) in dot-dashed line, and the invisibility condition of Eq. (8) drawn in dashed line, when the external medium has a permittivity *ε* = 1 (in black) and, for comparison, when *ε* = 4 (in yellow).

A possibility of tuning the invisibility window, as well as the window of the resonance peak, by modifying the chemical potential of the graphene coating that can be monitored by gate voltage seems to be very important for applications. The spectrum of *Q*_sca_ for a varying chemical potential *μ* is shown in Fig. 5(a), assuming a fixed core of radius *R* = 0.5 *μ*m and permittivity *ε*_*d*_ = 3.9. We observe that the peak-to-valley sinuosity governing the scattering spectrum is blueshifted for an increasing value of *μ*. As long as the chemical potential increases, the graphene conductivity also grows producing a shielding effect in the nanowire, which can be compensated by shifting the working wavelength to the near infrared. For example, the invisibility spectral band is 42.25 *μ*m wide when changing the chemical potential from 0.1 eV for *λ*_inv_ = 62.25 *μ*m to 1.0 eV for *λ*_inv_ = 20.00 *μ*m. One should notice, however, that the spectral width of the invisibility window, tuned by changing the graphene-shell chemical potential, depends on the dielectric core radius *R* and its permittivity, as well as *ε*. These parameters are responsible for cloaking activation at other frequencies. Therefore, the results presented in Fig. 5(a) are devoted specifically to *R* = 0.5 *μ*m, and *ε*_*d*_ = 3.9.

In Fig. 5(b) we present the scattering efficiency spectrum *Q*_sca_ for a graphene coated dielectric nanowire as a function of the core permittivity *ε*_*d*_, when immersed in air (*ε* = 1), while keeping a fixed *R* = 0.5 *μ*m, and *μ* = 0.5 eV. Negative values of the permittivity *ε*_*d*_ can be realized via semiconductor doping, e.g. n-InGaAs. In the spectral range of interest, dispersion of that semiconductor *ε*_*d*_(*ω*) can be very well described by the Drude model with the plasmon wavelength located at *λ*_*p*_ = 5.41 *μ*m^35^. The minimum of the scattering efficiency and the peak of the resonance both shift to longer wavelengths with an increase of the core permittivity. For the core permittivity *ε*_*d*_ only slightly higher than *ε*, the invisibility window significantly widens at the rate $d{\omega }_{{\rm{inv}}}/d{\varepsilon }_{d}$, which is notably higher than $d{\omega }_{{\rm{res}}}/d{\varepsilon }_{d}$, for the resonance peak window. For instance, the scattering minimum spans from 11.89 *μ*m to 16.62 *μ*m in the interval where *ε*_*d*_ changes from 1.5 to 2.0, whereas the variation of the resonant peak appears in the range of wavelengths from 26.09 *μ*m to 28.53 *μ*m. Moreover, when |*ε*_*d*_| < *ε*, the conducting graphene sheet can excite a resonant peak but the invisibility window vanishes. On the other hand, the maximum and the minimum of the curve virtually coincide for an epsilon-near-zero surrounding material. We point out that a non-negligible value of the imaginary part of *ε* imposes a correction of Eqs (7) and (8), thus preventing the peak-valley collapse. Inversely, high values of the environment permittivity enable to move the resonant peak further from the invisibility window, as depicted in Fig. 5(b) for *ε* = 4.

Note that nanostructured graphene metasurfaces potentially enable to accomplish a drastic reduction of the scattering efficiency in the case that *ε*_*d*_ < *ε*, even including conducting cylinders, by means of engineering its homogenized surface conductivity. For instance, an atomically thin metasurface composed of a periodic array of subwavelength graphene patches apparently exhibits a reconfigurable conductivity, whose imaginary part can reach arbitrary values from positive (at high frequencies) to negative (at low frequencies) in the THz spectrum by simply altering its geometric arrangement and Fermi energy of the graphene-based components^36^. When the patterned graphene metasurface is set over a subwavelength cylindrical scatterer, independently from its dielectric or conducting nature, a scattering cancellation is theoretically demonstrated with promising versatility in comparison to the homogeneous graphene monolayer^37,38^.

## Multiband scattering reduction with dimers

**Figure 6:**
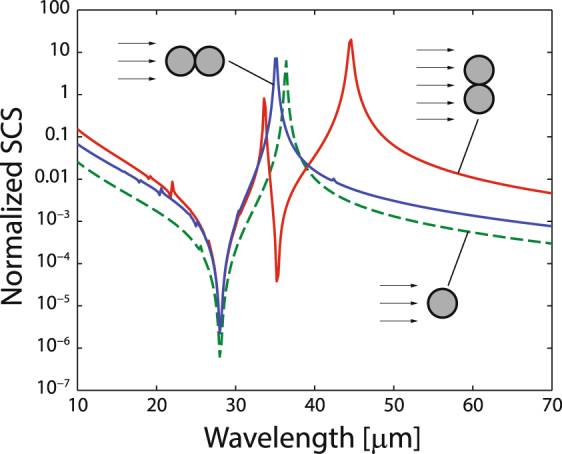
FEA-based numerical evaluation of the scattering cross section of a cluster of two graphene-coated nanowires of radius *R* = 0.5 *μ*m, core permittivity *ε*_*d*_ = 3.9, and graphene chemical potential *μ* = 0.5 eV, immersed in air. The SCS is normalized to the diameter of one nanowire, 2*R*. The nanowire dimer is oriented transversally (red solid line) and along (blue solid line) the propagation direction of the incident light. For comparison, we include the spectrum of *Q*_sca_ for a single nanoparticle (dashed green line).

Next, we analyze the scattering efficiency of clusters of two invisible graphene-coated nanocylinders in close proximity to each other. Since plasmonic coupling of the pair of particles is exclusively expected for incident TE^*z*^ polarized fields, our analysis is restricted to such case. In Fig. 6 we present the spectrum of the scattering cross section (SCS) that corresponds to nanowire dimers, conveniently normalized to the diameter of each particle $2R\,=\,1.0\,\mu {\rm{m}}$, assuming that their permittivity is ${\varepsilon }_{d}\,=\,3.9$ and that they are over-coated by a uniform-graphene shell with chemical potential $\mu \,=\,0.5\,{\rm{eV}}$. The pair of nanowires can be oriented either along the propagation direction of the incident plane wave, or perpendicularly. It is assumed that the axis of the cylinders is always in the *z*-direction. In the first case, a set of a peak resonance and a scattering minimum, found at ${\lambda }_{{\rm{res}}}\,=\,35.2\,\mu {\rm{m}}$ and ${\lambda }_{{\rm{inv}}}\,=\,28.0\,\mu {\rm{m}}$ respectively, stands out in the scattering lineshape which is comparable to the spectral pattern for a single graphene-coated nanowire, except maybe in a slight blue-shift of the resonance peak and an overall increased SCS magnitude. We point out that a secondary resonance might arise for larger particles where this plasmonic mode would be induced by the phase advance of the incident field propagating through the dimer^39^. For the case of two directly adjacent coated nanowires illuminated by light polarized along the interparticle *y* axis, two minima are identified at wavelengths ${\lambda }_{{\rm{inv1}}}\,=\,28.0\,\mu {\rm{m}}$, coinciding with the previously analyzed invisibility window, and ${\lambda }_{{\rm{inv2}}}\,=\,35.2\,\mu {\rm{m}}$ (here $R/{\lambda }_{{\rm{inv2}}}\,=\,1.42\times {10}^{-2}$). In addition, two resonant peaks are traced at ${\lambda }_{{\rm{res1}}}\,=\,33.6\,\mu {\rm{m}}$ and ${\lambda }_{{\rm{res2}}}\,=\,44.6\,\mu {\rm{m}}$.

The physical interpretation for the appearance of a couple of peaks relies on the strong coupling of the localized surface plasmon resonances of the individual particles^40^. The plasmonic response is altered due to a supplementary contribution to the total energy by a dipole-dipole coupled interaction^41^, together with higher-order multipolar contributions involved at the nanoscale proximity, with the result that a dramatic field enhancement occurs in the interparticle junction. And precisely such significant multipolar inputs are responsible of the collective resonant behavior of particle dimers. Importantly in our discussion, the near-field coupling of neighboring graphene-coated nanowires also induces a multiband reduction of the scattering cross section. In fact, such mode coupling with multiple resonance peaks and valleys has been both theoretically and experimentally reported for metallic particle dimers with a plethora of body shapes like spheres^42^, circular and ellipsoidal cylinders^43^, disks^44^, shells^45^, rods^46^, rings^47^, and others. The strong coupling between the graphene plasmons of each individual coated nanowire can be well described by a hybridization picture relying on the analogy between plasmons and the wave functions of simple quantum systems^48,49^.

**Figure 7:**
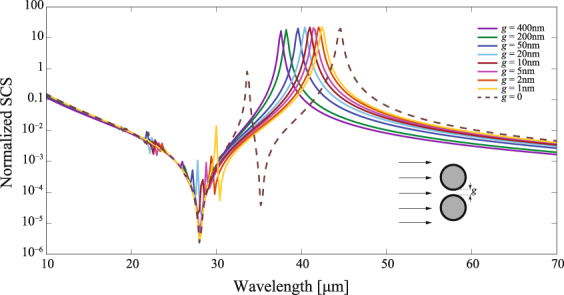
Scattering cross section, normalized to 2*R*, of a graphene-coated nanowire dimer oriented transversally to the propagation direction of the incident light. In the numerical simulations we again set $R\,\mathrm{=0.5}\,\mu {\rm{m}}$, ${\varepsilon }_{d}\,=\,3.9$, $\varepsilon \,=\,1$, and $\mu \,=\,0.5\,{\rm{eV}}$.

Figure 7 illustrates the scattering cross section for dimer coated nanowires having the same electromechanical characteristics as analyzed above but with a varying gap, here set perpendicularly to the direction of the incident field. The main invisibility wavelength remains practically unchanged at ${\lambda }_{{\rm{inv1}}}\,=\,28.0\,\mu$m, in fact corresponding to a frequency ${\omega }_{{\rm{inv}}}$ given in Eq. (8), for gaps ranging from *g* = 0 to *g* = 400 nm as graphically analyzed; meanwhile the main resonance peak is red-shifted as long as *g* decreases, a fact that also occurs in metallic dimers and discussed in detail in^50^. It is evident that gaps additionally enhance secondary resonances that in general increases the scattering cross section of the structure and thus partially suppress the invisibility effect. For instance, a small peak appears at $\lambda \,=\,26.2\,\mu$m for *g* = 200 nm which, on the other hand, has a limited impact on the scattering reduction observed in the vicinities of ${\lambda }_{{\rm{inv1}}}$. Importantly, this is critical for gaps around *g* = 20 nm where the secondary resonance peak lies near ${\lambda }_{{\rm{inv1}}}$; specifically at such a gap we find a peak at $\lambda \,=\,27.8\,\mu$m representing a SCS increment of around two orders of magnitude as compared with an ungapped dimer. Furthermore, shorter gaps cause a red shift far from the main invisibility window and, in fact, induce what looks like an intense Fano-like interference including a substantial reduction of the scattering efficiency in a secondary spectral range. A minimum with effective reduction in scattering efficiency is observed at ${\lambda }_{{\rm{inv2}}}\,=\,29.1\,\mu$m, ${\lambda }_{{\rm{inv2}}}\,=\,29.8\,\mu$m, and ${\lambda }_{{\rm{inv2}}}\,=\,30.4\,\mu$m when *g* = 5 nm, *g* = 2 nm, and *g* = 1 nm, respectively. In other words, a sharp decrease of the SCS is found within the spectral interval ranged from ${\omega }_{{\rm{res}}}$ to ${\omega }_{{\rm{inv}}}$, as estimated by Eqs (7) and (8), which can be spectrally controlled by the dimer gap *g* and is certainly advantageous for invisibility purposes. Remarkably, a sharp and abrupt change of the electromagnetic signature for a pair of graphene coated nanowires is evident as a conductive contact (that is *g* = 0) is formed.

**Figure 8:**
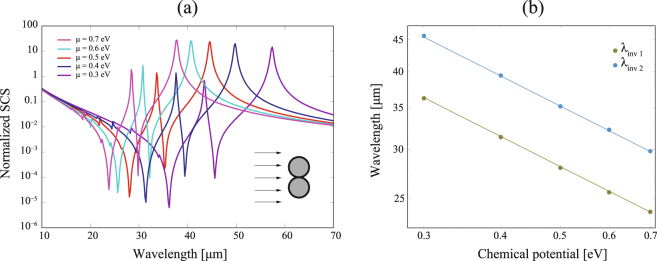
(**a**) The same as in Fig. 7 but the two nanowires have a zero gap and the graphene chemical potential *μ* varies. (**b**) Log-log plot of the primary invisibility wavelength ${\lambda }_{{\rm{inv1}}}$ and the the secondary invisibility wavelength ${\lambda }_{{\rm{inv2}}}$ showing a linear fitting in terms of the chemical potential in graphene.

Cylindrical plasmonic nanoshells with a dielectric core are supposed to have tunable resonance positions and a complex resonance structure, where decreasing shell thickness leads to a distinct redshift^51^. Here, the intrinsically two-dimensional structure of atomically-thin graphene requires a fundamentally different mechanism of fine-tune the spectral bands of resonance (and invisibility). Modification of the chemical potential $\mu$ of the graphene coating enables a significant shift not only of the invisibility and resonance frequencies of isolated coated nanowires, but also the multiband scattering reduction given in a nanowire dimer, as shown in Fig. 8(a). As we previously pointed out, the invisibility wavelength depends on the chemical potential as ${\mu }^{-\mathrm{1/2}}$, what can be concluded from Eq. (8). Now we observe that tunability based on the same dependence on $\mu$ is present in nanowire dimers for both the main invisibility wavelength, ${\lambda }_{{\rm{inv1}}}\,=\,{C}_{1}{\mu }^{-\mathrm{1/2}}$ where the proportional constant is estimated as ${C}_{1}\,=\,19.9\,\mu {\rm{m}}\,{{\rm{eV}}}^{\mathrm{1/2}}$, and the secondary invisibility wavelength ${\lambda }_{{\rm{inv2}}}\,=\,{C}_{2}{\mu }^{-\mathrm{1/2}}$ being ${C}_{2}\,=\,24.9\,\mu {\rm{m}}\,{{\rm{eV}}}^{\mathrm{1/2}}$. This is illustrated in Fig. 8(b) for nanowires of radius $R\,=\,0.5\,\mu {\rm{m}}$ and core permittivity ${\varepsilon }_{d}\,=\,3.9$ that are immersed in air.

**Figure 9:**
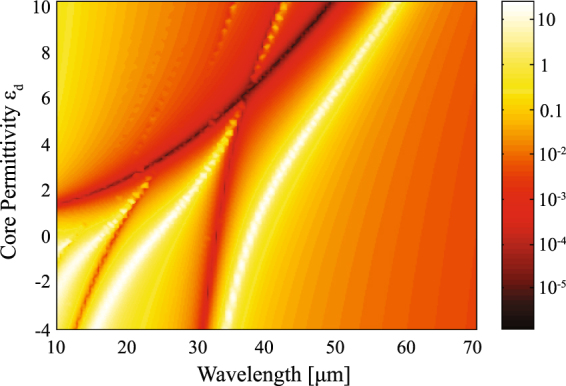
Scattering cross section normalized by $2R$ for a dimer nanowire with varying core permittivity. The rest of parameters are taken the same as in Fig. 7.

The existence of a multifrequency regime is also strongly determined by the permittivity of the nanowire core. Figure 9 represents the scattering cross section divided by $2R\,=\,1\,\mu$m for dimer cylinders of varying values of the parameter ${\varepsilon }_{d}$. Importantly, for ${\varepsilon }_{d}\, > \,6.2$ the dominant invisibility wavelength ${\lambda }_{{\rm{inv1}}}$ can be longer than the secondary invisibility band centered at ${\lambda }_{{\rm{inv2}}}$. Exactly at such limiting value ${\varepsilon }_{d}\,=\,6.2$, a single minimum of SCS is found at which ${\lambda }_{{\rm{inv1}}}$ and ${\lambda }_{{\rm{inv2}}}$ merge. For slightly lower permittivities of the nanowire core, the secondary minimum remains close to a resonance giving a shape of what remains a Fano interference. However, one should note that this is a wrong impression since the peak-valley pair is drifting apart as long as ${\varepsilon }_{d}$ decreases thus taking independent paths. On the other hand, a third invisibility band may be found at ${\varepsilon }_{d}\, < \,3.3$, set in the vicinities of an additional resonance peak. The increment of resonance peaks and scattering reduction bands is further enhanced for lower (in fact negative) values of the permittivity core. In this case, the nanowire core itself can exhibit a large number of resonances for the size here employed^52^, and due to the low absorption of graphene in the spectral range of interest, we can distinguish several peak positions caused by localized surface plasmons excitations of the graphene shell and the outer surface of the ${\varepsilon }_{d}$-negative core. The latter was also reported for bimetallic cylinder dimers in the visible^51^.

**Figure 10:**
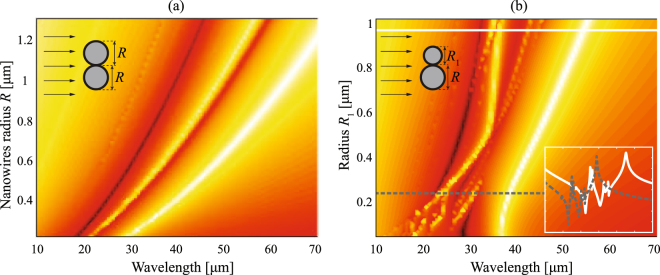
Scattering efficiency for a dimer nanowire with varying radius (**a**) for both nanowires, and (**b**) for one of the particles keeping fixed $R\,=\,0.5\,\mu$m. Inset: Log-plot spectrum of ${Q}_{{\rm{sca}}}$ for a dimer with ${R}_{1}\,=\,0.25\,\mu$m (dashed line) and ${R}_{1}\,=\,1\,\mu$m (solid line).

Now we analyze the influence of the cylinder radius to the scattering response of the dimer nanowires. Figure 10(a) shows the scattering efficiency when we modify the radius *R* simultaneously for both particles. By increasing the value of $R$, the shape of the SCS (here normalized by 4*R*) spectrum is essentially maintained but shifted to longer wavelengths. Therefore, both invisibility windows centered at ${\lambda }_{{\rm{inv1}}}$ and ${\lambda }_{{\rm{inv2}}}$ are also red-shifted accordingly. Within a good approximation, we might confirm that both wavelengths change in direct proportion to ${R}^{\mathrm{1/2}}$. This is not surprising since such behavior has been demonstrated for a single graphene-coated nanowire above (see Fig. 3). However, varying the radius of one of the cylinders, denoted as ${R}_{1}$, while the radius of the other component of the dimer is keeping fixed, $R\,=\,0.5\,\mu$m as shown in Fig. 10(b), reveals a completely different pattern. For instance if ${R}_{1}\,=\,0.25\,\mu$m, together with the two main resonances and invisibility bands we find a set of multiple secondary peaks and valleys. In fact a minimum is set just in the middle of the main peak of the lowest wavelength thus splitting it into two. Such complexity is repeated for other values of the radius as illustrated for ${R}_{1}\,=\,1\,\mu$m, however with an evident red-shifted spectrum. In conclusion, asymmetric dimers may exhibit an invisibility cloaking effect that is extended to numerous wavelengths.

Finally, it is worth noting that scattering cancellation can be expected to appear at more different frequencies when dealing with clusters formed by a higher number of graphene-coated nanowires. A previously proposed alternate multi-frequency cloaking mechanism is based on several graphene layers implementation at different gate charges^53^.

## Conclusions

We have demonstrated that the scattering behavior of a sufficiently thin graphene-coated nano-cylinder reminiscences a solid nanowire with a bulk conductivity equal to the ratio of graphene surface conductivity and the wire radius. In that case, some analytical expressions (see equations (6)–(8)) have been derived enabling the characterization of the spectral window for the invisibility cloaking. We have examined the possibility of tuning the invisibility window by monitoring the applied gate voltage to control the chemical potential of graphene. Moreover, we reveal that the spectral distribution of scattering efficiency, for the high index ratio between the core and the surrounding medium, approaches the well-known Fano resonance line shape. We propose an adequate multi-frequency invisibility mechanism that is based on a hybrid picture with clusters of several invisible graphene-coated nano-cylinders. Our findings can be straightforwardly applied to more complex graphene coating structures^37,54^. Finally, we would like to emphasize that the proposed scheme seems to be tunable enough to be implemented in ultra-thin reconfigurable cloaking devices. Within the far-infrared range of frequencies and applied to subwavelength scatterers, this undetectable cylindrical structure can be appropriate for sensing with dynamic operation bandwidth because the receiving field can efficiently reach its interior and inherently stimulate its electromagnetic response despite the strong suppression of its scattering efficiency. The occurrence of additional spectral minima by the excitation of coupled modes in nanowire dimers can cause a considerable increase of the gap field, capturing energy without radiate to the far field, that offers opportunities for different applications such as sensitive spectroscopy and novel nonlinear miniaturized systems.

## Methods

### Analytical considerations

To estimate analytically the scattering efficiency of the coated nanowire, we followed the Lorenz-Mie scattering method given in detail for instance in refs^10,14,18–20^.

#### TE^*z*^ polarization

Here we assume that the nanotube is illuminated by a TE^*z*^-polarized plane wave propagating along the $x$ axis, as illustrated in Fig. 1. The electromagnetic field of the incident plane wave may be set as

12 $${{\bf{H}}}_{in}=\hat{z}{H}_{0}\exp (ikx)=\hat{z}{H}_{0}\sum _{n=-\infty }^{+\infty }{i}^{n}{J}_{n}(kr)\exp (in\varphi ),$$

and ${{\bf{E}}}_{in}=i\nabla \times {{\bf{H}}}_{in}/\omega \varepsilon {\varepsilon }_{0}$, which reads as

13 $${{\bf{E}}}_{in}=-{E}_{0}\sum _{n=-\infty }^{+\infty }{i}^{n}[\hat{r}n\frac{{J}_{n}(kr)}{kr}+\hat{\varphi }i{J}_{n}^{^{\prime} }(kr)]\exp (in\varphi ),$$

where *r* and *ϕ* are the radial and azimuthal cylindrical coordinates, respectively, *H*_0_ is a constant amplitude, ${E}_{0}\,=\,{H}_{0}k/\omega \varepsilon {\varepsilon }_{0}$, ${J}_{n}(\cdot )$ is the Bessel function of the first kind and order $n$, ${k}_{0}\,=\,\omega /c$ is the wavenumber in the vacuum, and $k\,=\,{k}_{0}\sqrt{\varepsilon }$. Here the prime appearing in ${J}_{n}^{^{\prime} }(\alpha )$ denotes derivative with respect to the variable *α*. In Eq. (12) we used the Jacobi-Anger expansion of a plane wave in a series of cylindrical waves. The scattered electromagnetic field in the environment medium, $r > R$, may be set as ref.^16^

14 $${{\bf{H}}}_{sca}=\hat{z}{H}_{0}\sum _{n=-\infty }^{+\infty }{a}_{n}{i}^{n}{H}_{n}^{\mathrm{(1)}}(kr)\exp (in\varphi ),$$

and

15 $${{\bf{E}}}_{sca}=-{E}_{0}\sum _{n=-\infty }^{+\infty }{a}_{n}{i}^{n}[\hat{r}n\frac{{H}_{n}^{\mathrm{(1)}}(kr)}{kr}+\hat{\varphi }i{H}_{n}^{\mathrm{(1)}^{\prime} }(kr)]\exp (in\varphi ),$$

where ${H}_{n}^{\mathrm{(1)}}(\cdot )\,=\,{J}_{n}(\cdot )+i{Y}_{n}(\cdot )$ is the Hankel function of the first kind and order $n$, and the coefficients ${a}_{n}$ must be determined. The total magnetic field in the environment medium is simply ${{\bf{H}}}_{tot}\,=\,{{\bf{H}}}_{in}+{{\bf{H}}}_{sca}$. Finally, the electromagnetic field in the dielectric core of the coated cylinder ($r < R$) is expressed as

16 $${{\bf{H}}}_{d}=\hat{z}{H}_{0}\sum _{n=-\infty }^{+\infty }{b}_{n}{i}^{n}{J}_{n}({k}_{d}r)\exp (in\varphi ),$$

and

17 $${{\bf{E}}}_{d}=-\frac{{k}_{d}{H}_{0}}{\omega {\varepsilon }_{d}{\varepsilon }_{0}}\sum _{n=-\infty }^{+\infty }{b}_{n}{i}^{n}[\hat{r}n\frac{{J}_{n}({k}_{d}r)}{{k}_{d}r}+\hat{\varphi }i{J}_{n}^{^{\prime} }({k}_{d}r)]\exp (in\varphi ),$$

where the wavenumber ${k}_{d}={k}_{0}\sqrt{{\varepsilon }_{d}}$.

The Lorenz-Mie scattering coefficients ${a}_{n}$ and ${b}_{n}$, are determined by means of the proper boundary conditions. Due to the existence of graphene surface conductivity, the boundary conditions at *r* = *R* are given by ref.^55^ in order to ensure the continuity of tangential components of the electromagnetic field at the graphene shell:

18 $$\hat{\varphi }\cdot {{\bf{E}}}_{d}=\hat{\varphi }\cdot ({{\bf{E}}}_{sca}+{{\bf{E}}}_{in}),$$

19 $$\hat{z}\cdot {{\bf{H}}}_{d}=\hat{z}\cdot ({{\bf{H}}}_{sca}+{{\bf{H}}}_{in})+\hat{\varphi }\cdot {{\bf{E}}}_{d}\sigma ,$$

Since the electric field is assumed to be in the $xy$ plane, the *ϕ*-component of the effective conductivity has to be taken into account, only. These two equations can be written as

20 $$\frac{{k}_{d}}{{\varepsilon }_{d}}{b}_{n}{J}_{n}^{^{\prime} }({k}_{d}R)=\frac{k}{\varepsilon }[{J}_{n}^{^{\prime} }(kR)+{a}_{n}{H}_{n}^{\mathrm{(1)}^{\prime} }(kR)],$$

21 $${b}_{n}{J}_{n}({k}_{d}R)={J}_{n}(kR)+{a}_{n}{H}_{n}^{\mathrm{(1)}}(kR)-\sigma \frac{i{k}_{d}}{\omega {\varepsilon }_{d}{\varepsilon }_{0}}{b}_{n}{J}_{n}^{^{\prime} }({k}_{d}R)\mathrm{.}$$

Here, the coefficients with different index $n$ can be treated separately due to the linear independence of the multipolar components of the wave field. Finally, we obtain the analytical expressions for the scattering coefficients^14^:

22 $${a}_{n}=\frac{{k}_{d}{J}_{n}^{^{\prime} }({k}_{d}R)[\varepsilon {J}_{n}(kR)-i\frac{k\sigma }{\omega {\varepsilon }_{0}}{J}_{n}^{^{\prime} }(kR)]-k{\varepsilon }_{d}{J}_{n}({k}_{d}R){J}_{n}^{^{\prime} }(kR)}{k{H}_{n}^{\mathrm{(1)}^{\prime} }(kR)[{\varepsilon }_{d}{J}_{n}({k}_{d}R)+i\frac{{k}_{d}\sigma }{\omega {\varepsilon }_{0}}{J}_{n}^{^{\prime} }({k}_{d}R)]-{k}_{d}\varepsilon {H}_{n}^{\mathrm{(1)}}(kR){J}_{n}^{^{\prime} }({k}_{d}R)},$$

and

23 $${b}_{n}=\frac{k{\varepsilon }_{d}[{H}_{n}^{\mathrm{(1)}^{\prime} }(kR){J}_{n}(kR)-{H}_{n}^{\mathrm{(1)}}(kR){J}_{n}^{^{\prime} }(kR)]}{k{H}_{n}^{\mathrm{(1)}^{\prime} }(kR)[{\varepsilon }_{d}{J}_{n}({k}_{d}R)+i\frac{{k}_{d}\sigma }{\omega {\varepsilon }_{0}}{J}_{n}^{^{\prime} }({k}_{d}R)]-{k}_{d}\varepsilon {H}_{n}^{\mathrm{(1)}}(kR){J}_{n}^{^{\prime} }({k}_{d}R)}\mathrm{.}$$

#### TM^*z*^ polarization

Here we analyze the case of a graphene-coated nanowire illuminated by a TM^*z*^-polarized plane wave propagating along the $x$ axis by using again the Lorenz-Mie scattering method, and which was treated in ref.^10^. The incident electric field may then be written as

24 $${{\bf{E}}}_{in}=\hat{z}{E}_{0}\exp (ikx)=\hat{z}{E}_{0}\sum _{n=-\infty }^{+\infty }{i}^{n}{J}_{n}(kr)\exp (in\varphi )\mathrm{.}$$

The magnetic field is calculated as ${{\bf{H}}}_{in}=-i\nabla \times {{\bf{E}}}_{in}/\omega {\mu }_{0}$, which reads as

25 $${{\bf{H}}}_{in}={H}_{0}\sum _{n=-\infty }^{+\infty }{i}^{n}[\hat{r}n\frac{{J}_{n}(kr)}{kr}+\hat{\varphi }i{J}_{n}^{^{\prime} }(kr)]\exp (in\varphi ),$$

where ${H}_{0}\,=\,{E}_{0}k/\omega {\mu }_{0}$. In the environment medium, the scattered electromagnetic fields are now expressed as

26 $${{\bf{E}}}_{sca}=\hat{z}{E}_{0}\sum _{n=-\infty }^{+\infty }{c}_{n}{i}^{n}{H}_{n}^{\mathrm{(1)}}(kr)\exp (in\varphi ),$$

and

27 $${{\bf{H}}}_{sca}={H}_{0}\sum _{n=-\infty }^{+\infty }{c}_{n}{i}^{n}[\hat{r}n\frac{{H}_{n}^{\mathrm{(1)}}(kr)}{kr}+\hat{\varphi }i{H}_{n}^{\mathrm{(1)}^{\prime} }(kr)]\exp (in\varphi ),$$

where the scattering coefficients *c*_*n*_ must again be determined. Inside the nanowire, the fields finally are given as

28 $${{\bf{E}}}_{d}=\hat{z}{E}_{0}\sum _{n=-\infty }^{+\infty }{d}_{n}{i}^{n}{J}_{n}({k}_{d}r)\exp (in\varphi ),$$

and

29 $${{\bf{H}}}_{d}=\frac{{k}_{d}{E}_{0}}{\omega {\mu }_{0}}\sum _{n=-\infty }^{+\infty }{d}_{n}{i}^{n}[\hat{r}n\frac{{J}_{n}({k}_{d}r)}{{k}_{d}r}+\hat{\varphi }i{J}_{n}^{^{\prime} }({k}_{d}r)]\exp (in\varphi ),$$

Next applying the convenient boundary conditions at $r\,=\,R$, which simply are

30 $$\hat{z}\cdot {{\bf{E}}}_{d}=\hat{z}\cdot ({{\bf{E}}}_{sca}+{{\bf{E}}}_{in}),$$

31 $$\hat{\varphi }\cdot {{\bf{H}}}_{d}=\hat{\varphi }\cdot ({{\bf{H}}}_{sca}+{{\bf{H}}}_{in})-\hat{z}\cdot {{\bf{E}}}_{d}\sigma ,$$

leads to the analytical expressions for the scattering coefficients *c*_*n*_ and *d*_*n*_. Then, they are given by

32 $${c}_{n}=\frac{{k}_{d}{J}_{n}(kR){J}_{n}^{^{\prime} }({k}_{d}R)-{J}_{n}({k}_{d}R)[k{J}_{n}^{^{\prime} }(kR)+i{\mu }_{0}\sigma \omega {J}_{n}(kR)]}{k{H}_{n}^{\mathrm{(1)}^{\prime} }(kR){J}_{n}({k}_{d}R)-{H}_{n}^{\mathrm{(1)}}(kR)[{k}_{d}{J}_{n}^{^{\prime} }({k}_{d}R)-i{\mu }_{0}\sigma \omega {J}_{n}({k}_{d}R)]},$$

and

33 $${d}_{n}=\frac{k[{H}_{n}^{\mathrm{(1)}^{\prime} }(kR){J}_{n}(kR)-{H}_{n}^{\mathrm{(1)}}(kR){J}_{n}^{^{\prime} }(kR)]}{k{H}_{n}^{\mathrm{(1)}^{\prime} }(kR){J}_{n}({k}_{d}R)-{H}_{n}^{\mathrm{(1)}}(kR)[{k}_{d}{J}_{n}^{^{\prime} }({k}_{d}R)-i{\mu }_{0}\sigma \omega {J}_{n}({k}_{d}R)]}\mathrm{.}$$

Note that in the limit $R\to 0$ the zero-th order dominates the scattering properties of the graphene-coated cylinder. In such a case, the scattering efficiency is correctly estimated as ${Q}_{sca}\,=\,\mathrm{4|}{c}_{0}{|}^{2}/kR$. In addition we might simplify the dominant scattering coefficient by using the lowest orders of the series expansion such as ${J}_{0}(x)\approx 1-{x}^{2}\mathrm{/4}$ and ${Y}_{0}(x)\approx [\,\mathrm{ln}(x\mathrm{/2)}+\gamma \mathrm{]2/}\pi$, where $\gamma$ is the Euler’s constant. This finally gives us

34 $${c}_{0}=\frac{i\pi }{4}{(kR)}^{2}\frac{{\varepsilon }_{d}-\varepsilon +i2\sigma /{\varepsilon }_{0}\omega R}{\varepsilon }\mathrm{.}$$

**Figure 11:**
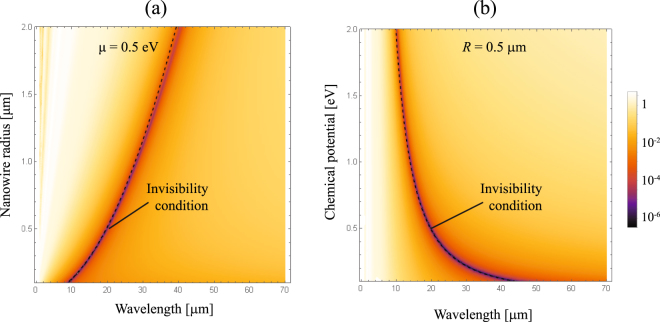
Contour plot of ${Q}_{sca}$ for graphene coated cylinders illuminated by a TM^*z*^-polarized plane wave, when (**a**) the radius *R* varies, maintaining fixed ${\varepsilon }_{d}\,=\,3.9$ and *μ* = 0.5 eV. (**b**) The chemical potential *μ* is varied, keeping ${\varepsilon }_{d}\,=\,3.9$ and *R* = 0.5 *μ*m. We also represent the curve for the invisibility condition ${\omega }_{{\rm{inv}}}^{TM}\,=\,\sqrt{2}{\omega }_{{\rm{inv}}}$, drawn in dashed line, when the external medium has a permittivity *ε* = 1.

**Figure 12:**
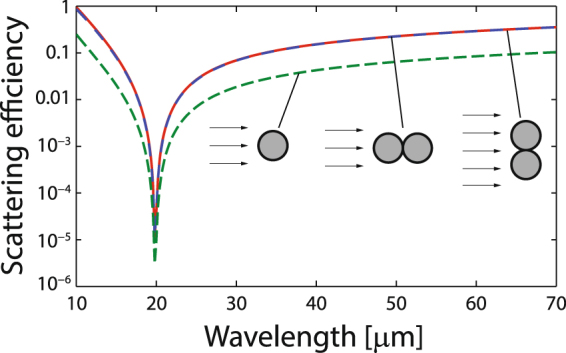
FEA-based numerical evaluation of the scattering efficiency, as performed in Fig. 6 but using a TM^*z*^ polarized incident plane wave.

As expected, no resonance can be found under TM^*z*^ polarized fields. On the other hand, the cylinder behaves like a conducting uncoated particle of bulk conductivity $2\sigma /R$, which is double when compared with the effective conductivity experienced under TE^*z*^ polarization. Accordingly, the invisibility frequency is blue-shifted by a factor $\sqrt{2}$, as illustrated in Fig. 11. In fact, the scattering properties and consequently the invisibility frequency will be preserved even for dimmer nanowires, as shown in Fig. 12. For TM^*z*^ polarized wave fields, the estimation of an effective permittivity *ε*_eff_ as discussed just below Eq. (11) for single coated particles also can be clearly applied to clusters, thus supporting the results of our numerical simulations.

### Numerical simulations

The problem of multiple scattering by an arbitrary configuration of nonoverlapping parallel cylinders is essentially a boundary-value problem which has been considered by many authors^56–58^. Here, the SCS is numerically evaluated by using COMSOL Multiphysics, which is a commercially available solver of the Maxwell’s equations based on the finite element analysis (FEA). The circular active domain has a radius of $4\lambda$ and is bounded by perfectly matched layers in order to reach perfect absorption at its perimeter. An adaptive meshing with a maximum grid size of 20 nm at every inner boundary and subsequent refining of the mesh were chosen, specially in the dimer interspacing.
